# CD161 identifies polyfunctional Th1/Th17 cells in the genital mucosa that are depleted in HIV-infected female sex workers from Nairobi, Kenya

**DOI:** 10.1038/s41598-017-11706-y

**Published:** 2017-09-11

**Authors:** Geneviève Boily-Larouche, Kenneth Omollo, Julianna Cheruiyot, Jane Njoki, Makobu Kimani, Joshua Kimani, Julius Oyugi, Julie Lajoie, Keith R. Fowke

**Affiliations:** 1 Department of Medical Microbiology and Infectious Diseases, University of Manitoba, Manitoba, Canada; 2 Department Medical Microbiology, University of Nairobi, Nairobi, Kenya; 3 Kenya AIDS Control Project, University of Nairobi, Nairobi, Kenya; 4 Department of Community Health Science, University of Manitoba, Manitoba, Canada

## Abstract

CD161 identifies a subset of circulating Th17 cells that are depleted in the blood and gut of HIV-infected individuals. In the female reproductive tract (FRT), the pattern of CD161 expression on CD4^+^ cells remains unknown. Here, we characterized CD161 expression in the FRT of Kenyan female sex workers (FSW). Compared to the blood, CD161^+^CD4^+^ T cells were enriched in the FRT of uninfected FSWs. These cells were depleted in FRT of HIV-infected FSWs. Cervical CD161^+^ cells harboured an activated phenotype (CD69, CD95, HLA-DR) with elevated expression of tissue-homing markers (CCR6, β7 integrin) and HIV co-receptor (CCR5). Mitogen-stimulated production of IL-17 confirmed the Th17 commitment of CD161^+^CD4^+^ T cells in the FRT with a predominance of polyfunctional Th1/Th17 cells. Here, we showed that the expression of CD161 on CD4+T cells is modulated at the FRT, but still identified a highly activated cellular subset, which differentiates into pro-inflammatory Th1/Th17 cells, expresses multiple HIV susceptibility markers and are depleted in HIV-infected individuals. The use of CD161 as a biomarker of HIV targets in the FRT reduces the need for functional assessment of cells and could have important implications in better understanding HIV pathogenesis and Th17 fate in the FRT of high-risk women.

## Introduction

In 2015, fifty-six percent of new HIV infections in sub-Saharan Africa were among women and this number continues to grow disproportionally^1^, reinforcing the importance of bringing scientific attention to women’s health. The female reproductive tract (FRT) is characterized by the presence of several innate mucosal factors that exert effective defences against HIV entry^2^. However, this protective role can be disrupted by inflammation-driven breaches in the mucosal defence, which facilitates HIV penetration into the genital mucosa. Factors driving or sustaining inflammation at the FRT are believed to promote HIV infection through the activation and recruitment of CD4^+^ T cells^3, 4^. However, we do not yet fully understand the landscape of HIV targets in the genital tract and the factors regulating their recruitment and activation.

CD161 is a type II transmembrane glycoprotein, a member of the C-type lectin family and a recognized biomarker of the T helper 17 lineage^5–7^. IL-17-producing cells differentiate from CD161 precursors and maintain the expression of CD161 throughout their life cycle, comprising ∼20% of the circulating^7, 8^ and ∼60% of the colonic lamina propria CD4^+^ T lymphocytes^5, 9^. CD161^+^CD4^+^ T cells exhibit an effector memory phenotype with a transcriptional profile including Th17 signature genes such as *IL-23R, RORγT, IL-17A, IL-22, IL18R* and *CCR6* ^5, 7, 10^. Th17 are involved in pathogen defence and maintenance of barrier integrity and are highly enriched in mucosal compartments, including the FRT where they primarily localize in the endo- and ecto-cervix^11, 12^.

CD161^+^CD4^+^ T cells are enriched in a variety of inflammation-driven auto-immune diseases, including inflammatory bowel disease^5^, Rheumatoid arthritis (RA)^13–15^, and juvenile idiopathic arthritis^16^, suggesting pathogenic functions. They are recruited to the inflamed tissue where they sustain the local inflammatory response by inducing the production of immune mediators by epithelial cells and fibroblasts^5, 15^. The pro-inflammatory nature of this subset makes it an optimal candidate for initiating HIV infection at the FRT either by promoting inflammation at the genital tract or by acting as targets to amplify the infection since Th17 cells are preferential targets for HIV in the FRT^12, 17^. To our knowledge, the pattern of CD161 expression remains unknown in the FRT and the nature and phenotype of these cells have never been explored. Understanding the dynamic of CD161^+^CD4^+^ T cells recruitment and function at the FRT may provide important insights into HIV susceptibility.

In this study, we characterised the CD161-expressing CD4^+^ T cells in the FRT of female sex workers (FSWs) from Nairobi, Kenya. First, we defined the phenotypic and functional profile of the cervical and circulating CD161^+^ and CD161^-^ fractions, and we investigated their potential as HIV target cells and driver of inflammation in the FRT of HIV-uninfected and chronically infected FSWs.

## Results

### CD161^+^CD4^+^ lymphocytes are enriched in the female genital tract and comprise activated cells with high expression of the HIV co-receptor C-C chemokine receptor 5 (CCR5)

**Figure 1:**
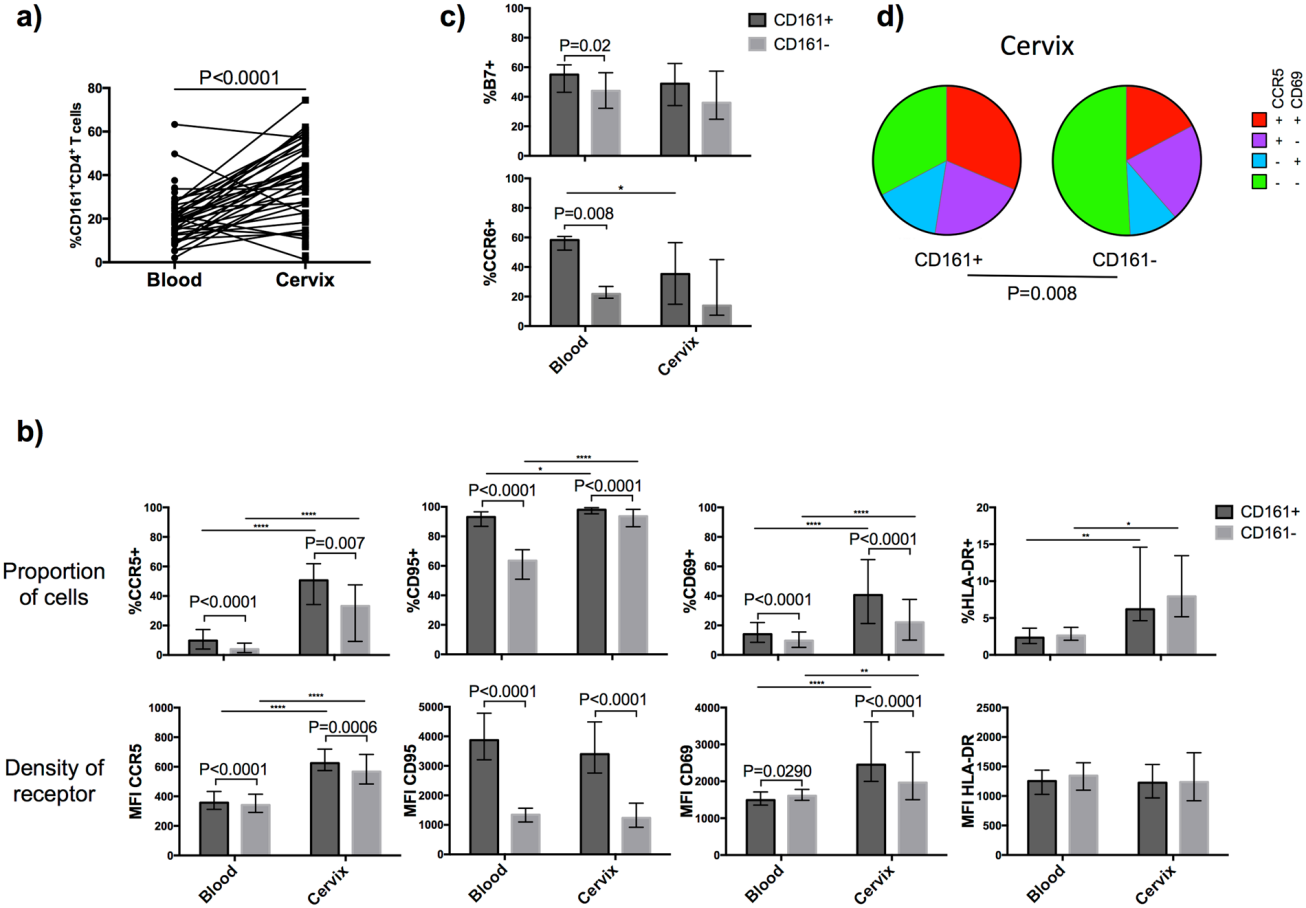
Proportion and phenotype of CD161^+^ and CD161^−^ T helper cells in blood and cervix of HIV-uninfected female sex workers (FSWs). (**a**) Proportion of CD161^+^CD4^+^ T cells in blood and cervix, difference between compartments were assessed using Wilcoxon Matched Pair Rank. (**b**) Phenotype of CD161^+^ and CD161^−^ fraction. Proportion of cells expressing CCR5, CD69, HLA-DR and CD95, (**c**) proportion of homing markers β7 and CCR6 expressing cells among the blood and cervical CD161^+^ and CD161^−^ fraction of CD4^+^ lymphocytes. Data are reported as median, interquartile range (IQR) and difference between fractions and compartments were calculated using Two-Way Anova, when significant, Wilcoxon signed-rank test was used to compare fractions. *P < 0.05, **P < 0.01, ***P < 0.001, ****P < 0.0001. (**d**) Co-expression of CD69 and CCR5 on cervical CD161^+^ and CD161^−^ fraction, Wilcoxon Rank test was used to assess the difference between pie chart.

Using flow cytometry, we defined the phenotype of the CD161^+^ and CD161^−^ fraction among CD4^+^ T cells of sixty-nine HIV-negative FSWs from Nairobi, Kenya. Socio-demographic, clinical and behavioural characteristics are presented in Table 1. We compared the phenotype and proportion of both populations between blood and cervical compartments to assess whether CD161^+^CD4^+^ T cells are recruited to the FRT and if their recruitment alters their phenotype. We observed that CD161^+^CD4^+^ T cells were enriched in the FRT of HIV-negative FSWs compared to blood. In the cervical compartment, a median proportion of thirty-eight percent of the total CD4^+^ T cells were expressing CD161 (median: 38, interquartile range; IQR: 21–53), which was 2 times higher than in the blood compartment (median: 18, IQR: 13–25) (P < 0.0001) (Fig. 1a). The CD161^+^ fraction displayed a higher proportion of activated cells (expressing CCR5, CD69 or CD95, Fig. 1b) and a greater proportion expressed mucosal homing markers (CCR6 expression was 3 fold higher than CD161^−^ fraction, P = 0.008; integrin β7 expression was 1.3 fold higher, P = 0.02) (Fig. 1c). A similar phenotype profile was observed among HIV-positive women (see Supplementary Fig. S1). Preferential recruitment and subsequent retention of the CD161^+^ fraction in the cervix could explain the enrichment observed in the FRT.

**Table 1:** Socio-demographic characteristics.

	HIV-negative	HIV-positive	P value
n	69	16	
Self-declared years of sex work			
Median (IQR)	7 (2–11)	7 (4.75–10.75)	0.2587*
Age			
Median (IQR)	35 (31–40)	38 (35–40)	0.0582*
Clients in the last 7 days			
Median (IQR)	5 (2–8)	9 (6–20)	0.0008*
Self-reported condom use			
100%	75% (49/65)	81% (13)	0.7508^#^
<100%	25% (16/64)	19% (3)	
Regular partner/client			
Yes	61% (41/67)	62% (10/16)	1.000^#^
Marital status			
Not married, not living with a man	64% (42/66)	60% (9/15)	0.9987^#^
Not married, living with a man	8% (5/66)	7% (1/15)	
Married, not living with a man	29% (19/66)	33% (5/15)	
Married, living with a man	0	0	
CD4 count at baseline			
Median (IQR)	921 (729–1083)	719 (543–758)	0.0066*
Days since the last menses^&^			
Median (IQR)	8 (6–9)	10 (8–12)	0.0257*
Phase of the menstrual cycle at baseline^&^			
Follicular	89% (60/67)	69% (11/16)	0.0487^#^
Vaginal douching in the last 3 days			
Yes	37% (22/59)	44% (4/9)	0.7232^#^
Nugent Score at baseline			
Normal	46% (32/69)	40% (6/16)	0.0573^#^
Intermediate	25% (17/69)	53% (8/16)	
Bacterial Vaginosis	28% (19/69)	7% (1/16)	
Presence of a sexually transmitted infection at some point during the follow-up			0.5783^#^
*Neisseria gonorrhoea*	3% (2/69)	6% (1/16)	
*Chlamydia trachomatis*	4% (3/69)	0% (0/10)	
*Trichomonas vaginalis*	1% (1/69)	0% (0/16)	
Treponema pallidum (Syphilis)	1% (1/69)	0% (0/69)	
Candidiasis at some point during the follow up	61% (42/69)	63% (10/16)	0.9040^#^

^*^Student T tests were used when normally distributed, Mann-Whitney were used when the distribution was not normal. ^#^Chi-square analysis.

^&^Calculated according to the self-reported dates of last menses.

CD69 regulates tissue retention and identifies tissue-resident subsets^18^ while CCR5 is a chemotactic mediator directing migration of inflammatory cells to the tissue. We, and others^11^, have observed that the proportion of CCR5 and CD69 expressing cells was enhanced in the genital compartment (Fig. 1b) underlying the possible role of these molecules in migration and retention of CD4^+^ lymphocytes into the mucosa. Notably, in the cervix of HIV-negative women, the CD161^+^ fraction was enriched in CCR5^+^CD69^+^ double positive cells (Fig. 1d) (P = 0.02). Interestingly, co-expression of these markers is the strongest correlates of HIV entry in the cervical cells^19^. Thus, the homing capacity and the memory nature of the CD161^+^CD4^+^ T cells supports the preferential migration of this activated subset from the periphery to the cervix and their subsequent cervical retention to potentially act as HIV targets.

### Cervical CD161^+^CD4^+^ T cells are enriched in IL-17A secreting cells but Th17 commitment is not exclusive to the CD161^+^ fraction in the FRT

**Figure 2:**
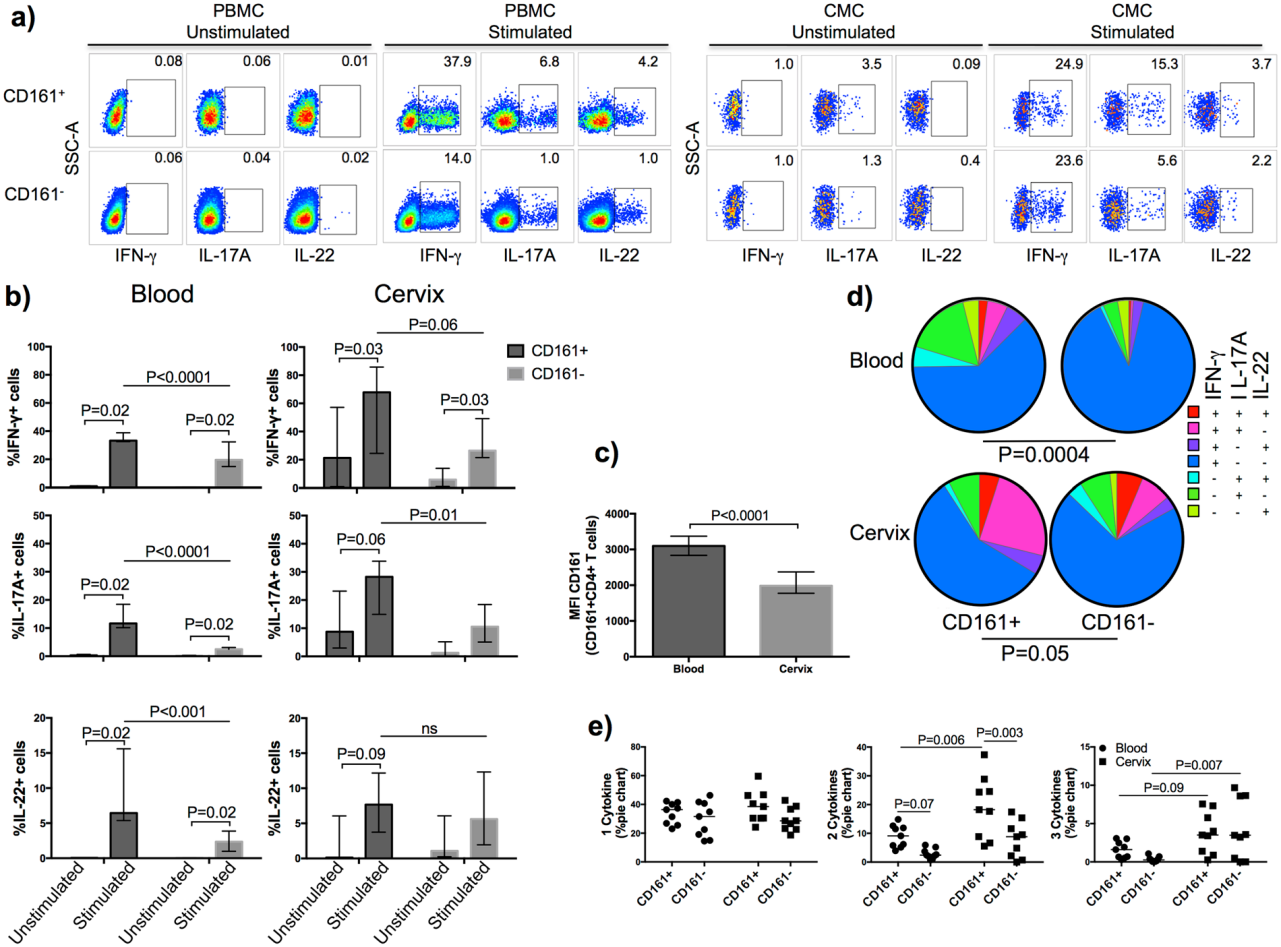
Polyfunctionality of cervical CD161^+^ and CD161^−^ T helper cells, as measured by the co-expression of IL-17A, IL-22, and/or IFN-γ. (**a**) Gating strategy. (**b**) Proportion of bulk cervical and systemic CD4^+^ T cells producing the cytokines IL-17A, IFN-γ and IL-22 in HIV- uninfected after PMA/ionomicin stimulation (median, IQR). Repeated Measures Two-Way Anova and Sidak’s multiple comparison post-test to report the P value adjusted for multiplicity were used to calculate the difference between fractions and compartments, *P < 0.05, **P < 0.01, ***P < 0.001, ****P < 0.0001. (**c**) Density of cell surface expression of CD161 as reported with the median fluorescence intensity (MFI; median, IQR) between the blood and cervical CD4^+^ T cells. (Paired t test). (**d**) Polyfunctional profile of cervical CD161^+^ and CD161^−^ T helper cells, as measured by the co-expression of IL-17A, IL-22, and/or IFN-γ. The cervical CD161^+^ fractions were more polyfunctional than the CD161^−^ fraction in the blood and cervix, and cervical cells were more polyfunctional than blood cells. Each possible combinations of IL-17A, IL-22 and IFN-γ production by CD161^+^ and CD161^−^ cells is shown; box plots depict the interquartile range (Wilcoxon Rank test was used to assess the difference between pie chart). (**e**) Proportion of cells within the CD161^+^ and CD161^−^ CD4^+^ T cell populations producing one, two or three cytokines in blood and cervix (Repeated Measures Two-Way Anova and Sidak’s multiple comparison post-test with P value adjusted for multiplicity).

Th17 cells are derived from CD161 blood precursors^7, 20^ and it was shown that virtually all IL-17A and IL-22-secreting cells were contained within the CD161^+^ fraction, even though only a small proportion of them become Th17 cells^5–7, 14, 16, 20, 21^. We further characterized the PMA/ionomycin-stimulated cytokine secretion profile of CD161^+^CD4^+^ T cells in the blood and cervix of ten HIV-negative women and investigated whether the secretion of IL-17A and IL-22 was also exclusively contained within the CD161^+^ fraction in the FRT (see Supplementary Fig. S2, and Fig. 2a for gating strategy). Because Th17 cells are enriched in the cervical compartment^11^, we hypothesized that the enhanced Th17 lineage commitment observed in the FRT was the result of an enrichment in CD161^+^ precursors and that CD161 would be an efficient marker to easily identify Th17 cells.

As reported before^11, 12^, we observed an increased proportion of IL-17A^+^ (P = 0.02) cells in the FRT compared to the blood compartment (Fig. 2b). Furthermore, the expression of IL-17A was elevated within the CD161^+^ fraction (Fig. 2b). The median proportion of PMA/ionomycin-stimulated IFN-γ and IL-17A-secreting cells in the cervical CD161^−^ fraction was 2.5 times higher than in the CD161^−^ fraction (IFN-γ: CD161^+^; median: 68, IQR: 25–86 vs CD161^−^; median: 26, IQR: 22–49, p < 0.05 and IL-17A: CD161^+^; median: 28, IQR: 15–34, CD161^−^; median: 11, IQR: 5–18, P < 0.05). Surprisingly, in the cervix, IL-17A and IL-22 expression was not exclusive to the CD161^+^ fraction (Fig. 2b). To assess whether the Th17 commitment observed in the CD161^−^ fraction could be due to the down-modulation of CD161 at the cell surface following migration to the FRT, we compared CD161 density at the cell surface by measuring the Median Fluorescence Intensity (MFI) of the CD161^+^ fraction. We found a 1.6 times decrease in MFI of CD161 on CD161^+^ cervical cells compared to circulating cells (blood; 3096, IQR: 2833–3370 and cervix; 1985, IQR: 1777–2373) (P < 0.0001) (Fig. 2c).

Th17 cells are highly plastic and can undergo a profile change under inflammatory conditions. We assessed the polyfunctionality of the CD161^+^ and CD161^−^ fractions through Boolean gating and witnessed a difference in the cytokine secretion profiles (Fig. 2d). The diversity in IL-17A, IL-22 and IFN-γ combinations was increased in the CD161^+^ fraction for both compartments (blood, P = 0.0004, cervix, P = 0.05) (Fig. 2d). The cervical compartment was significantly more polyfunctional than the blood compartment with both fractions harbouring elevated proportion of cells secreting three cytokines simultaneously (Fig. 2e). Combination of two cytokines was significantly higher in the CD161^+^ fraction (Fig. 2e) and in the cervix when compared to blood (p = 0.006). A larger proportion of the cervical CD161^+^ cells differentiated in Th1/Th17 cells, with dual-production of IL-17A^+^IFN-γ^+^ (Fig. 2d). This suggests that migration to the FRT results in increased polyfunctionality of CD4^+^ T cells, with further differentiation of the CD161^+^ precursors into Th1/Th17.

In the cervical compartment, as in the blood, all IL-17-expressing cells also express CCR6^12, 22^. Interestingly, when assessing the co-expression of these two well-known markers of Th17, a third of the IL-17-expressing CD4^+^ lymphocytes were contained in the CCR6^+^CD161^−^ in the FRT while in the blood, only a tenth of IL-17-expressing cells were contained in this fraction while the majority were contained in the double positive fraction (see Supplementary Fig. S3a,b). In the cervix, the functional profile of the CCR6^+^CD161^+^ fraction was highly similar to the CCR6^+^CD161^−^ (Supplementary Fig. S3c). Down-modulation of CD161 after migration to the FRT could, in part, explain the presence of IL-17 secreting cells within the CD161^−^ fraction. Nevertheless, we cannot exclude that the unique microenvironment of the FRT drives the differentiation of Th17 subsets from CD161^−^ cells.

### The cervical environment modulates the expression of CD161

Given that the fate of Th17 cells can be shaped by inflammatory conditions^9, 20^ and that Th17 cells play an important role in regulating immunity against fungal and bacterial pathogens in the FRT^23^, we hypothesized that alteration of the genital microenvironment and/or bacterial infections would be in part responsible for the unique pattern of CD161 expression observed in the FRT. Using a linear-mixed effect model, we evaluated whether common bacterial sexually transmitted infections (STIs), Bacterial Vaginosis (BV) infection and inflammatory markers were associated with CD161 modulation in the cervical compartment of HIV-uninfected (n = 69) FSWs followed longitudinally for 6 visits. The cervical concentrations of inflammatory markers were measured in the CVL of women using a panel of 18 pro-inflammatory cytokines and chemokines (see Supplementary Table S1). Overall, five women were diagnosed with *Neisseria gonorrhoeae, Chlamydia trachomatis*, *Trichomonas vaginalis* or *Treponema pallidum*, resulting in eleven events of infections (Table 1). BV infection was detected in thirty-two women during the follow-up period. Crude size effect and P values are reported in Supplementary Table S2. P values adjusted for age and menstrual cycle phase are also reported.

**Figure 3:**
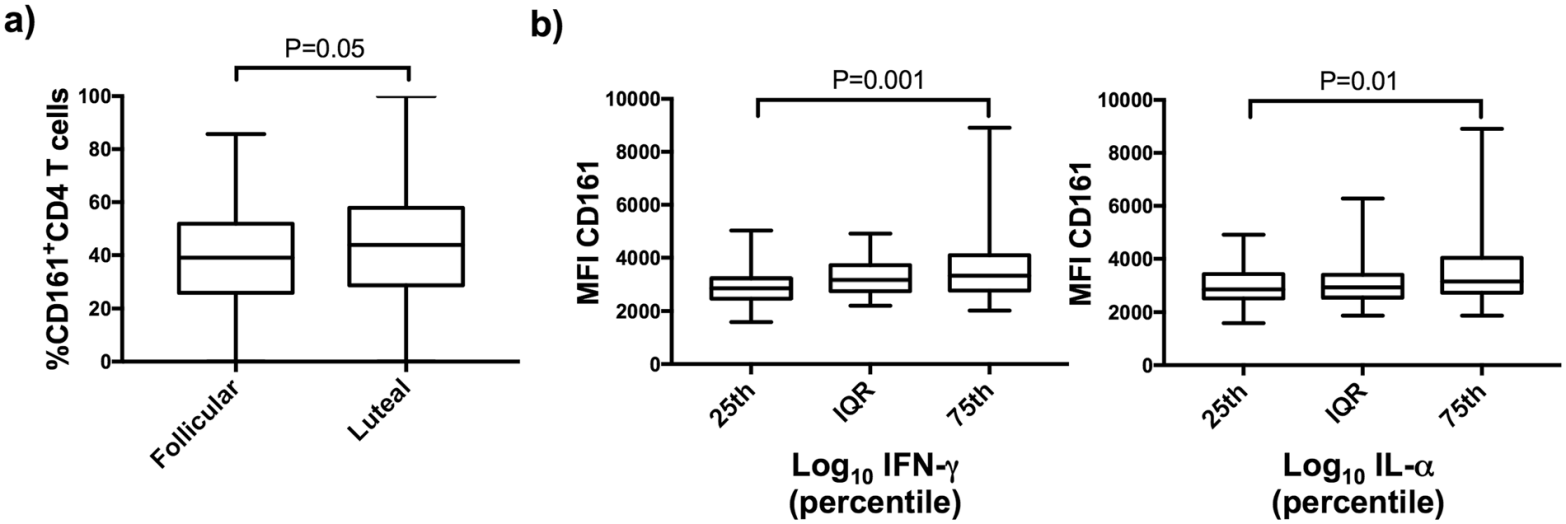
Impact of the genital microenvironment of the proportion and expression of CD161 on CD4^+^ T cells in the FRT of HIV-uninfected FSWs. (**a**) Comparison of the proportion of CD161^+^CD4^+^ T cells in the FRT between phases of the menstrual cycle using a linear regression model (reference follicular phase). Comparison of the CD161 Median Fluorescent Intensity (MFI) gated on CD161^+^CD4^+^ T cells between quartiles of log_10_ transformed cervical concentration of IFN-γ (**b**) and IL-1α (**c**) among HIV-uninfected FSWs using a linear regression (reference 25^th^ percentile). Results are presented as whisker boxes showing the median and interquartile range (IQR); whiskers extend between the minimum and maximum values. P values adjusted for age, or age and menstrual cycle phase are reported.

Overall, we did not observe any significant correlation between the presence of BV or STIs infection, the cervical concentrations of inflammatory markers or the proportion of CD161^+^CD4^+^ T cells in the FGT of HIV-uninfected FSWs (See Supplementary Table S2). An elevated proportion CD161^+^CD4^+^ T cells in the cervical compartment during the luteal phase was however identified (size effect adjusted for age: 3.9%, P = 0.05) (Fig. 3a), implying that hormonal regulation may have more impact on the recruitment of CD161^+^CD4^+^ T cells in the genital compartment than bacterial infection.

When assessing the impact of the genital microenvironment on the modulation of CD161 expression, we observed that the highest expression of CD161 on CD4^+^ T cells was observed amongst women with elevated cervical concentrations of IFN-γ and IL-1α (Fig. 3b) (size effect IFN-γ: 465 representing an increase in 16% in MFI per category, P = 0.001, size effect IL-1a: 422, 14% increase in MFI per category, P = 0.01). Hence, bacterial infection or inflammatory markers did not seem to directly influence the recruitment of CD161^+^CD4^+^ T cells in the cervical compartment, but elevation of IFN-γ and IL-1α contributes to maintaining high level of CD161 cell surface expression in the FRT. Interestingly, despite the fact that BV infection did not directly influence CD161 expression, in our study (see Supplementary Table S3), like in other studies^24^, BV infection was associated with high level of pro-inflammatory markers, including IFN-γ and IL-1α in the FRT.

### Preferential depletion of cervical CD161^+^CD4^+^ T cells is observed in the FRT of HIV-positive FSWs

We have established that CD161^+^CD4^+^ T cells displayed an ideal phenotype for HIV entry, with high density of CCR5 and CD69 and enriched in IL-17-secreting cells. Given the reported role of this subset in gut inflammation and other inflammatory related diseases, we hypothesized that CD161 could serve as a marker to identify a cervical sub-population that may be a key target for HIV infection. Blood CD161^+^CD4^+^ T cells are permissive to HIV infection and depletion of this population has been observed in individuals chronically infected by HIV^25^. We compared the proportion of *ex vivo* CD161^+^CD4^+^ T cells between untreated chronically HIV-infected FSWs (n = 16) and HIV-uninfected FSWs (n = 69).

**Figure 4:**
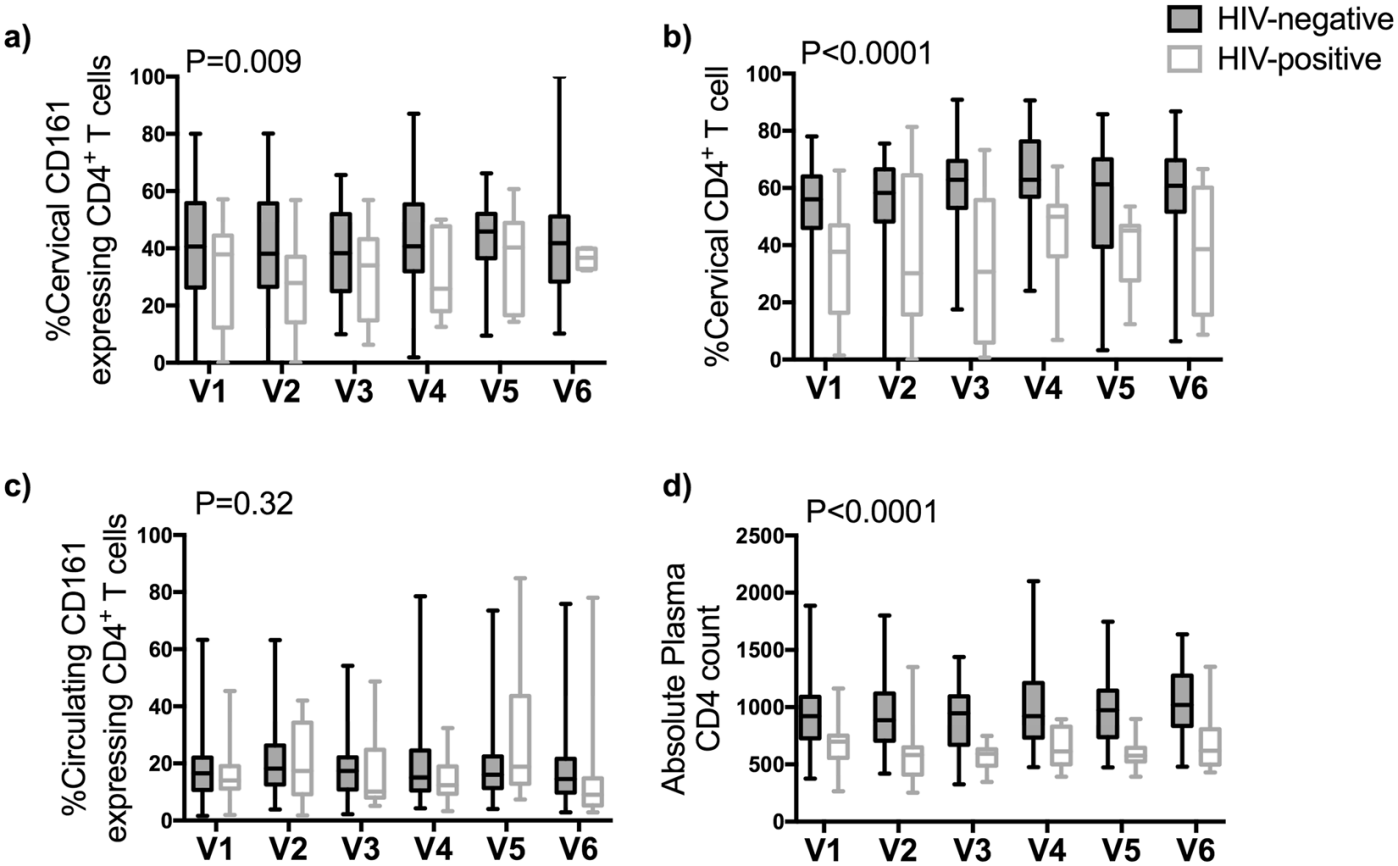
Comparison of the proportion of (**a**) CD161^+^ cells in the cervical CD4^+^ T cell compartment, (**b**) CD4^+^ T cells in the cervical CD3^+^ T cell compartment, (**c**) CD161^+^ cells in the systemic CD4^+^ T cell compartment, and (**d**) absolute plasma CD4 count between HIV-negative (reference) and HIV-positive FSWs at each visit using a linear regression. Boxes show the median, interquartile range (IQR); whiskers extend between the minimum and maximum values. Age and menstrual cycle adjusted P values are shown.

We observed a diminution of the mean proportion of cervical CD161^+^CD4^+^ T cell in HIV-infected FSWs compared to the mean proportion among HIV-negative FSWs (P = 0.009)(Fig. 4a) in addition to an overall depletion of the proportion of cervical CD4^+^ T cells in the CD3^+^ compartment (Fig. 4b, P < 0.0001). This specific depletion was unique to the FRT as in the blood, the proportion of circulating CD161^+^CD4^+^ T cells was similar between HIV-infected and uninfected FSWs despite the obvious depletion of CD4 lymphocytes (Fig. 4c,d). This observation is in agreement with previous observation that Th17 cells in the FRT are depleted during HIV infection^11, 17^.

## Discussion

Susceptibility to HIV infection is highly dependent on the degree of cervical inflammation^26^. Sex hormones, genital infections and bacterial microflora all regulate the genital microenvironment and its downstream effect on the recruitment of HIV targets^4, 24, 27, 28^. Inflammation in the FRT is the result of a complex interplay between epithelial and immune cells and a comprehensive portrait of this inflammatory landscape is still lacking. The documented role of CD161-expressing CD4^+^ T lymphocytes in pathogenic auto-immunity^5, 13–15^ and their lineage commitment to Th17 cells, make them a likely candidate to enhance HIV susceptibility through modulating the inflammatory environment, or more directly, by acting as HIV targets at the portal of entry.

We explored, for the first time, the phenotype and inflammatory profile of CD161^+^CD4^+^ lymphocytes at the FRT. We report an enrichment of the CD161^+^ fraction among the cervical T helper cells in the FRT and this finding aligns closely with the reported increase in IL-17-expressing cells previously observed^11, 12^. We also observed that the CD4^+^ T cells identified by CD161 in the FRT differ in nature and function from the blood compartment in terms of Th17 differentiation capacity and cytokine secretion profile. The fate of Th17 cells can be shaped by inflammatory conditions allowing distinct patterns of plasticity^9, 20^. Here, we report that redistribution of the CD161^+^ fraction to the FRT induces further differentiation of the Th17 cells into Th17/Th1 cells that display high inflammatory capacity and co-production of IL-17A and IFN-γ. A similar fate has been reported for CD161^+^ cells isolated from inflamed tissue in individual suffering from multiple sclerosis, arthritis or RA where co-expression of IL-17A and IFN-γ is the hallmark of inflammatory pathologies^13–15, 29^. In our study, CD161^+^ cells co-expressing IL-17A and IFN-γ were enriched in the FRT of HIV uninfected women, and their proportion was associated with the luteal phase of the menstrual cycle but not cervical inflammation or bacterial infections, suggesting that in the FRT, this subset may not display inflammatory features but be part of the FRT normal environment.

CD161 expression in the FRT also seems to be altered by its migration to the FRT, with a general surface down-modulation of the marker. Even though most IL-17A^+^ cells were restricted to the CD161^+^ fraction, a significant proportion of IL-17^+^ cells were contained in the CD161^−^ fraction, underlying an important difference in terms of Th17 origin and fate in the FRT. CD161^+^CD4^+^ lymphocytes acquire the ability to produce IL-17A under exposure to IL-1β and IL-23 and become mature Th17 cells. Then, in presence of IL-12, Th17 cells acquire the ability to produce IFN-γ in addition to IL-17A (Th17/Th1) and can further become “nonclassical” Th1 that have loss IL-17 production^9^. The nature of antigen priming also highly influences the fate of Th17 cells. *Candida albicans*–specific cells produce IFN-γ and IL-17 under the regulation of IL-1β while *Staphylococcus aureus*-specific Th17 cells produce IL-17 and IL-10^22^. Partial down-modulation of CD161 following bacterial stimulation has previously been reported^30^. In our study, we observed that cervical IL-17^+^ cells were harbouring more frequently a Th1/Th17 phenotype than blood IL-17^+^ cells and a lower cell surface expression of CD161 on cervical CD4^+^ T cells compare to blood cells. This argues for an enhanced capacity for CD161^+^CD4^+^ precursors to differentiate into Th1/Th17 cells and non-classical Th1 under the unique FRT microenvironment, with a gradual loss of CD161 expression under homeostatic conditions. The factors that drive this presumed CD161 down-modulation are still to be explored, however our data would suggest that neither bacterial infections nor inflammatory cytokines drove this phenomenon.

We observed, for the first time, a depletion of the CD161^+^CD4^+^ T cells in the cervical compartment of HIV-infected participants. McKinnon *et al*. previously identified a subset of CD4^+^ T cells expressing α4β7, CCR5, IFN-γ and IL-17A, preferentially binding gp120 and depleted during HIV infection^11^. Others have shown that Th17/Th1 cells display high expression of CCR5, *in vitro* permissibility for HIV infection, and support viral persistence in HIV-infected individuals^31^. All of these characteristics are contained within the subset of cells that express CD161 and support the hypothesis that the CD161^+^ fraction is the ideal target for HIV. Moreover, when comparing FSWs with different degrees of HIV susceptibility (HIV-exposed seronegative vs HIV susceptible women), we did not observed a significant difference in cervical proportion of CD161^+^CD4^+^ T cells, suggesting that low FRT proportion of CD161^+^CD4^+^ T cells may not explain intrinsic difference in HIV susceptibility, but rather may be the consequence of the infection. Altogether, this suggests that the ideal phenotype for HIV entry displayed by the CD161^+^ fraction (i.e. CCR5^+^) is also translated into a preferential depletion of this subset at the portal of entry.

Identifying Th17 cells in the FRT using the cytokine signature represents a technical challenge given the low cell yield, the fragility of cervical cells and the plasticity of Th17. Here, we showed that the expression of the CD161 is modulated following the migration to the FRT, allows for the identification of a highly activated cellular subset, which differentiates into polyfunctional pro-inflammatory Th1/Th17 cells, expresses multiple HIV susceptibility markers and identifies a subset of cells depleted during HIV infection. This has important implication to better understand HIV pathogenesis and Th17 fate in the FRT.

## Methods

### Study participants

FSWs were recruited from the Pumwani Sex Worker Cohort between May 2013 and September 2014 at the Majengo Clinic in Nairobi, Kenya. Sixty-nine HIV-negative and sixteen antiretroviral therapy (ART) naive chronically HIV-infected FSWs were included in the study. At the time of the study the CD4 counts of the HIV infected women were above the threshold for initiation of ART as standard of care in Kenya (CD4 < 350). The women active in sex work for more than seven years were defined as HIV-exposed seronegative (HESN) and were considered to have reduced susceptibility to HIV infection^32, 33^. The seronegative women practising sex work for less than three years were considered susceptible to HIV acquisition and called new HIV-negatives^32, 33^. Because no difference was observed between groups in term of CD161^+^ cells phenotype and function, all HIV-negative women were analysed together. Institutional Review Boards from Kenyatta National Hospital (Nairobi, Kenya) and the University of Manitoba (Winnipeg, Canada) approved the study and experimental protocols, and informed written consent was obtained from all participants. All experiments were performed in accordance with relevant guidelines and regulations. Women not using hormonal contraceptive were recruited during the follicular phase of the menstrual cycle (3–5 days post menses) and followed every two weeks for three months. A clinical, demographic and behavioural questionnaire was completed at every visit, followed by a clinical examination and sample collection. Peripheral blood for CD4 counts (FACScount) and vulvovaginal swabs were collected at every visit. Nugent’s criteria to score Bacterial Vaginosis (BV) and presence of candidal pseudohyphae or spores were confirmed by microscopy and syphilis infection by serology. *Trichomonas vaginalis* was diagnosed from vaginal swabs using the In-Pouch kit (Biomed Diagnostics, USA). Urine samples were collected for PCR detection of *Neisseria gonorrhea* and *Chlamydia trachomatis* (Roche Amplicor kits, USA). HIV serology using rapid test (Determine, Inverness Medical, Japan) was performed at the first and last visits, and positive status were confirmed by ELISA (Vironostika, Biomerieux, France). No women seroconverted during the study.

### Sample collection and processing

Blood and cervical cytobrushes were obtained for all participants for *ex vivo* phenotyping. Given the low yield of cervical mononuclear cells (CMC) obtained through cytobrushes, functional studies were carried using fresh cells from ten new HIV-negative participants. The ten participants were FSWs recruited at the same clinic during the ongoing study. For these ten women, cervical and blood samples were collected once and a clinical, demographic and behavioural questionnaire was completed. Cervical samples were collected and processed as described in Juno *et al*.^34^. Two milliliters of sterile 1x PBS saline was used to wash the ectocervix and collected from the posterior fornix. Samples were kept on ice and transferred to the laboratory within 2 hours. The samples were centrifuged and stored at −70 °C. Cervical cells were collected from an endocervical cytobrush and ectocervical spatula scraping. Cells were collected under speculum examination by inserting the cytobrush and the scraper into the endocervical os, rotating 360°, and immediately placing both the cytobrush and scraper into the 50 ml falcon tube containing 5 ml of PBS.

Blood tubes were collected with venipuncture using heparin as anticoagulant and peripheral blood mononuclear cells (PBMC) were isolated by Ficoll density gradient.

### PBMC and cervical cells stimulation and Flow cytometry

PBMC and cervical cells were washed with 1x PBS-2% FBS and stained for *ex vivo* phenotyping. Cells were scraped off the cytobtush and spatula and filtered through a 100 μM nylon cell strainer to a fresh 50 mL falcon tube, centrifuged and washed in RPMI 1640 (Invitrogen), and resuspended in PBS for staining with Live-dead-PE-Texas-Red (Invitrogen), CD3-PEcy5, CD4-FITC, CCR5-V450, CD161-APC, CD69-PECy7, HLA-DR-APC-H7, CD8-V500, (BD Biosciences; Biosciences, Mississauga, ON, Canada). The proportion of HIV target cells among CD4 T cells was determined by flow cytometry. Given the low cell yield normally obtained from the cervix, we analyzed only CMC samples with ≥100 events in the live CD3^+^ gate. Data were acquired on a BD LSR II cytometer (BD Biosciences) and analyzed with FlowJo Software (version 10.0.7; Tree Star, Ashland, OR, USA).

The CD4^+^CD161^+^ T cells function was assess through PMA/ Ionomycin stimulation. PBMC (10^6^) and cervical cells were stimulated for five hours using 50 ng/ml of PMA and 1 ng/ml Ionomycin (Sigma, USA) in RPMI solution (RPMI 1640 media containing 100 U/ml penicillin, 100 mg/ml streptomycin, Invitrogen) and stained for dead cells. Cells were stained for intracellular cytokine expression. PBMC (10^6^) and cervical cells were stained with PE.Cy5-CD3, FITC-CD4, V500-CD8, PE-CD95, APC. H7-HLA-DR, APC-CD161, Alexa700-CD45RA, V450-CCR5, PE. Cy7-CD69, PE-CF594-CCR7 (BD Biosciences, USA), or Far Red-Live Dead discriminant (Invitrogen, USA) for *ex vivo* phenotyping. Cell were stained prior to fixation and permeabilization using PE. Cy5-CD3, APC-CD161, PE-Cy7-CCR6, or PeCy7-CD8, Alexa-700-CD4, and BrillantViolet421-TCRvalpha7.2-B421 and after with PE-Dazzle-IL-17A, PE-IL-22 and FITC-IFN-γ (BD Biosciences).

### Cytokines/chemokines detection

CVL samples were centrifuged and stored at −70 °C. Cervical concentrations of the pro-inflammatory cytokines (Supplementary Table S1) were measured by Milliplex (Millipore, Germany) and analyzed on the BioPlex-200 (Bio-Rad, Canada) as in Lajoie *et al*.^35^.

### Statistical analysis

D’Agostino & Pearson normality test was used to test the assumption that the data sets were distributed following Gaussian distribution. Two-way Anova and non-parametric Wilcoxon signed-rank test were performed using Prism 6.0 f (GraphPad Software, USA) to compare the difference between compartments or fractions. Repeated Measures Two-Way Anova and Sidak’s Multiple Comparisons Post-test were used to calculate the difference between fractions and compartment and P value adjusted for multiplicity are reported. Cell polyfunctionality was assessed using SPICE version 5.35 (National Institute of Allergy & Infectious Diseases, USA). We used a linear mixed-effect model to estimate the effect of each variable (age, menstrual cycle phase, BV status, STI, BV, Candidiasis, phase of menstrual cycle, age, inflammatory status, log_10_ transformed concentration of inflammatory markers) on the density and proportion of CD161 on CD4^+^ T cells (outcome variable). Variance components were estimated using residual maximum likelihood. Correlation coefficients are reported to estimate the size effect. A positive coefficient represents an increase in the outcome unit for each increase of a unit of the variable while a negative coefficient represents a negative correlation. Analyses were performed using STATA version 13.1 (StataCorp, USA). Crude P values are reported in addition to P values adjusted for age and/or menstrual cycle phase. P value were considered significant < 0.05.

### Data availability

The datasets generated and analysed during the current study are available from the corresponding author on reasonable request.

## Electronic supplementary material

Supplementary Information
