# Supplementary material for: CD161 identifies polyfunctional Th1/Th17 cells in the genital mucosa that are depleted in HIV-infected female sex workers from Nairobi, Kenya
Source: Sci Rep. 2017 Sep 11;7:11123. doi: 10.1038/s41598-017-11706-y (PMC5593931; doi:10.1038/s41598-017-11706-y)
Supplement: Supplementary file 1 — Supplementary Information [file 41598_2017_11706_MOESM1_ESM.pdf]

Supplementary Information

CD161 identifies polyfunctional Th1/Th17 cells in the genital mucosa that are depleted in HIV-infected female sex workers from Nairobi, Kenya

Geneviève Boily-Larouche[1], Kenneth Omollo[2], Julianna Cheruiyot[3], Jane Njoki[3], Makobu Kimani[3], Joshua Kimani[3], Julius Oyugi[2,3], Julie Lajoie[1,2], Keith R. Fowke[1,2,4],*.

[1]Department of Medical Microbiology and Infectious Diseases, University of Manitoba; [2]Department Medical Microbiology, University of Nairobi; [3]Kenya AIDS Control Project, University of Nairobi; [4]Department of Community Health Science, University of Ma-nitoba

*keith.fowke@umanitoba.ca

Supplementary Figure S1

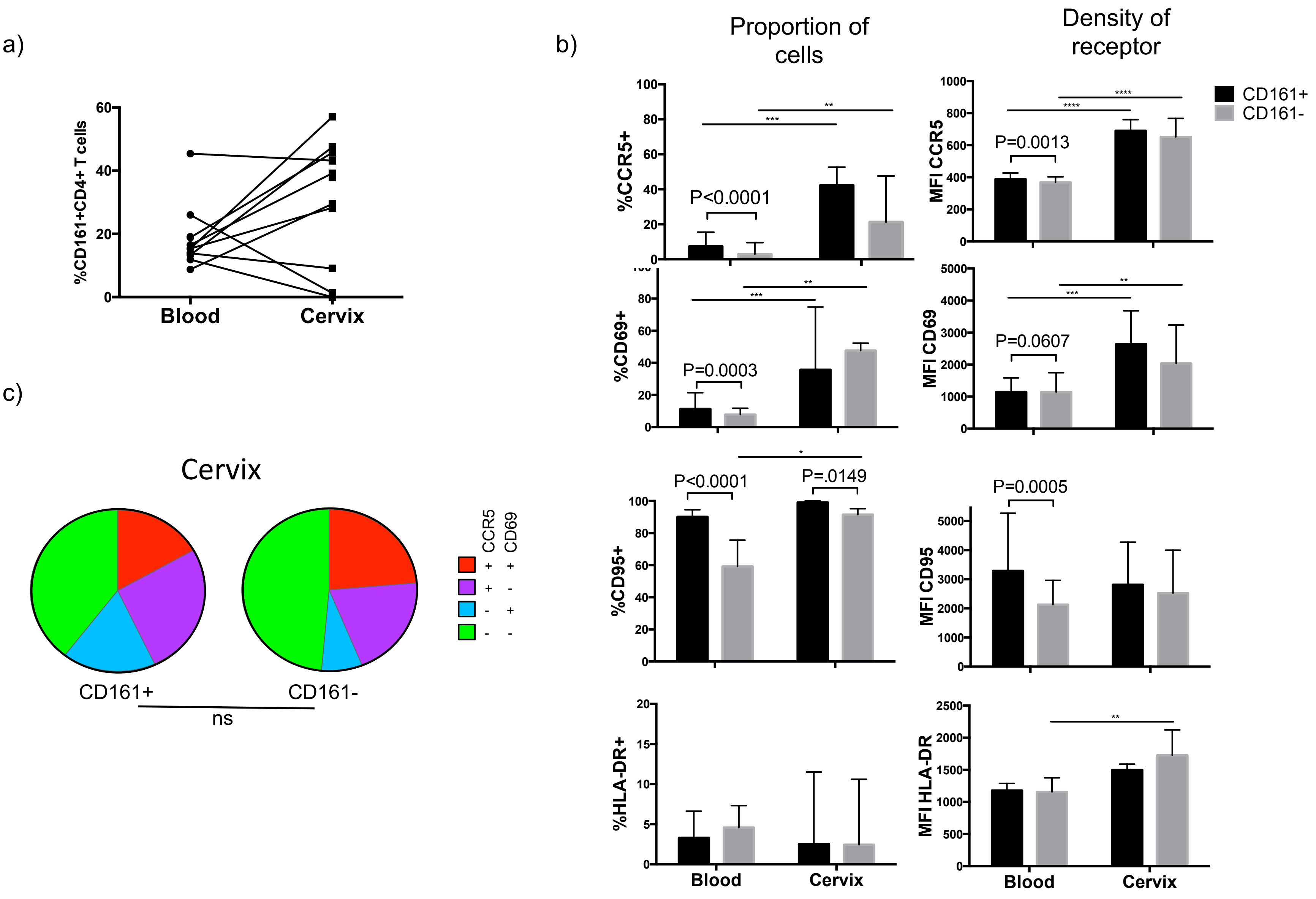

Supplementary Figure S1. Proportion and phenotype of $CD161^+$ and $CD161^-$ T helper cells in blood and cervix of HIV-infected female sex workers (FSWs). a) Proportion of $CD161^+CD4^+$ T cells in blood and cervix, difference between compartments were assessed using Wilcoxon Matched Pair Rank. b) Phenotype of $CD161^+$ and $CD161^-$ fraction. Proportion of cells expressing CCR5, CD69, HLA-DR and CD95, c) proportion of homing markers $\beta 7$ and CCR6 expressing cells among the blood and cervical $CD161^+$ and $CD161^-$ fraction of $CD4^+$ lymphocytes. Data are reported as median, interquartile range (IQR) and difference between fractions and compartments were calculated using Two-Way Anova, when significant, Wilcoxon signed-rank test was used to compare fractions. *p<0.05, **p<0.01, ***p<0.001, **** p<0.0001. c) Co-expression of CD69 and CCR5 on cervical $CD161^+$ and $CD161^-$ fraction, Wilcoxon Rank test was used to assess the difference between pie chart.

Supplementary Figure S2

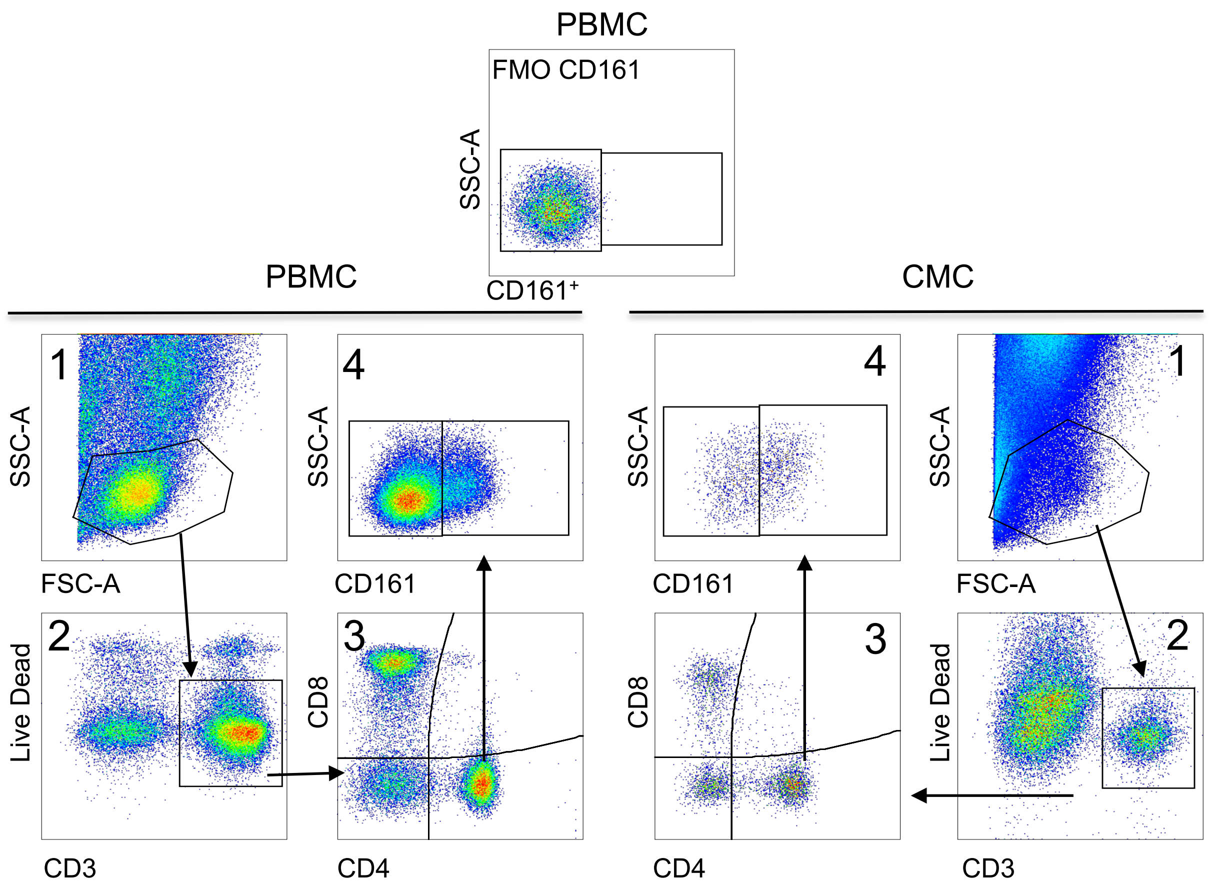

Supplementary Figure S2. Gating strategy to characterize polyfunctional mucosal and systemic T helper cells co-expressing CD161. For each peripheral blood mononuclear cell (PBMC) and cervical mononuclear cell (CMC) samples,100,000 total events were collected and gated to identify lymphocytes (FSC-A vs SSC-A, 1), select for live $CD3^+$ cells (2), subdivide the $CD3^+$ gate into $CD4^+$ and $CD8^+$ T cell subsets (3), and define the $CD161^+$ and $CD161^-$ fraction (4). The Fluorescent Minus One (FMO) control for the CD161 gate is shown in the upper panel. Cells expressing IFN-γ, IL-17A, and IL-22 were further gated within the $CD161^+$ and $CD161^-$ fraction (Figure 2a).

Supplementary Figure S3

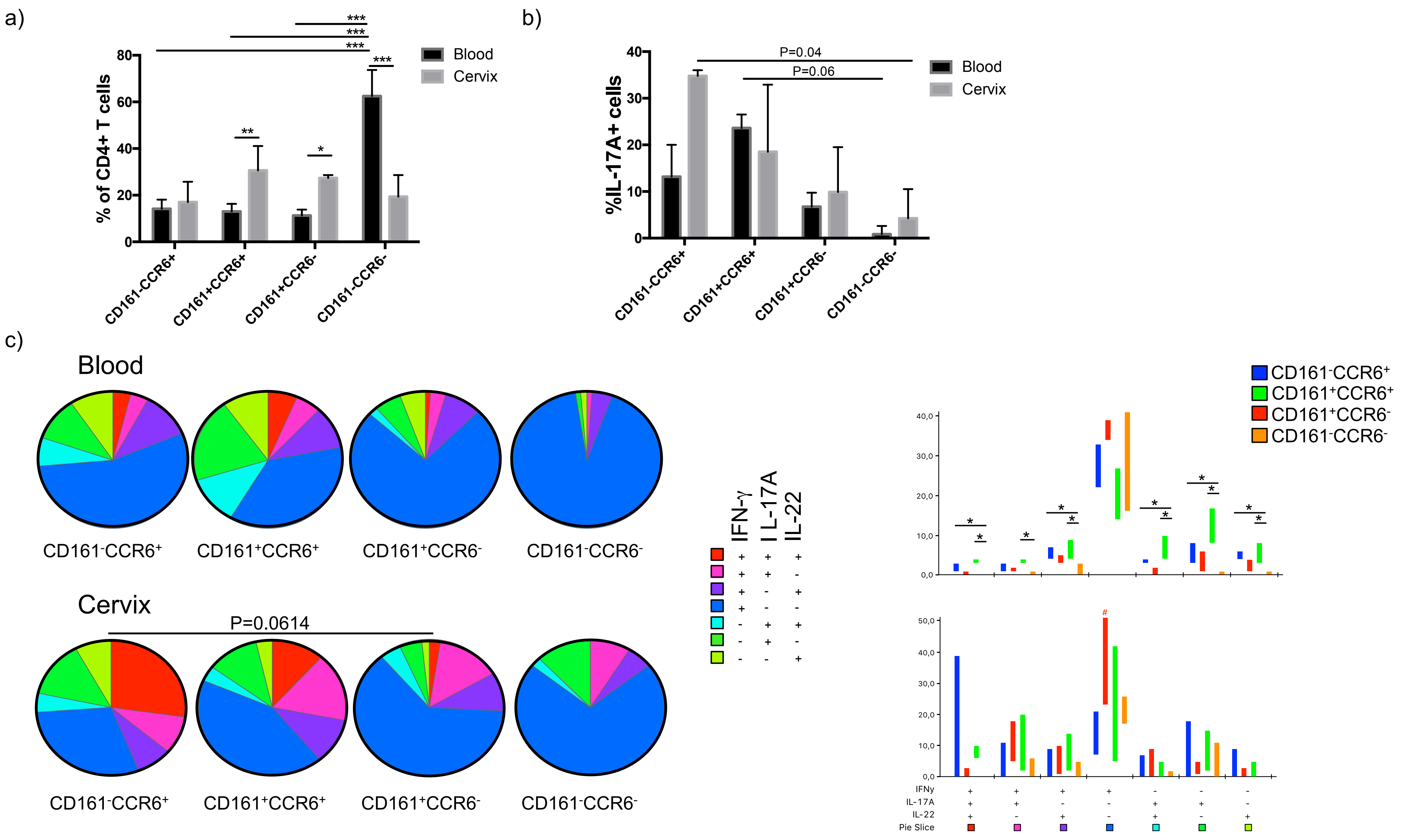

Supplementary Figure S3. Polyfunctionality of mucosal T helper cells co-expressing CD161 and CCR6, as measured by the co-expression of IL-17A, IL-22, and/or IFN-γ. a) Distribution of $CD4^+$ T cells in blood and cervix co-expressing CD161 and CCR6. b) Proportion of $CD4^+$ T cells co-expressing CD161 and CCR6 among the IL-17-producing cells. Repeated Measures Two-Way Anova and Sidak's multiple comparisons post-test to report the p-value adjusted for multiplicity and calculate difference between fractions and compartments, and Mann-Whitney was used to compare fractions, *$p<0.05$, **$p<0.01$, ***$p<0.001$, ****$p<0.0001$. c) The polyfunctional profile of the $CD4^+$ T cells co-expressing CD161 and CCR6 from blood and cervix with each possible combination of IL-17A, IL-22 and IFN-γ production by $CD161^+$ and $CD161^-$ cells is shown; box plots depict the interquartile range. Wilcoxon Rank test was used to assess the difference between pie charts.

Supplementary Table S1: Cervical concentrations of inflammatory markers in HIV-negative FSWs from Nairobi, Kenya. Assessment of the analyte variation over time (6 visits) using linear regression and showing coefficient and confidence intervals (CI 95%). At the exception of IL-1RA, all markers did not significantly fluctuate during the study period.

		HIV-negative (n=62)			
Predictors	>LLD	Median (IQR) °	Coefficient (CI 95%)	P-value#	P-value*
IFN-g	35.8%	-0.40 (-0.40, 0.61)	0.02 (-0.008, 0.05)	0.166	0.069
IL-12p70	55.8%	0.60 (-0.52, 0.97)	0.02 (-0.02, 0.06)	0.377	0.089
sCD40L	34.1%	0.41 (0.41, 0.72)	0.003 (-0.01, 0.02)	0.724	0.447
IL-10	18.9%	-0.26 (-0.26, -0.26)	-0.0007 (-0.03, 0.03)	0.959	0.639
IL-17A	34.7%	-0.46 (-0.46, 0.63)	0.006 (-0.03, 0.04)	0.704	0.376
IL-1a	93.5%	2.03 (1.59, 2.57)	0.02 (-0.01, 0.05)	0.193	0.260
IL-1b	73.4%	1.10 (-0.40,1.74)	0.02 (-0.03, 0.07)	0.449	0.381
IL-2	30.9%	-0.30 (-0.30, 0.56)	0.009 (-0.02, 0.04)	0.543	0.294
IL-8	97.8%	2.84 (2.39, 3.27)	-0.01 (-0.05, 0.03)	0.597	0.566
IP10	81.3%	1.73 (1.24, 2.27)	0.0009 (-0.04, 0.04)	0.961	0.980
MCP1	91.6%	1.50 (0.91, 2.09)	-0.02 (-0.06, 0.02)	0.378	0.899
MIP-1a	62.1%	0.81 (0.16, 1.20)	-0.007 (-0.04, 0.02)	0.636	0.753
MIP-1b	80.8%	1.08 (0.71, 1.35)	0.01 (-0.01, 0.04)	0.331	0.088
TNF-a	23.3%	-0.46 (-0.46, -0.46)	-0.02 (-0.05, 0.008)	0.167	0.430
IL1RA	100%	4.81 (4.45, 5.25)	0.03 (0.002, 0.06)	0.039	**0.023**
MIG	71.5%	2.23 (0.71, 2.76)	-0.01 (-0.06, 0.03)	0.557	0.559
MIP-3a	37.1%	-0.10 (-0.10, 1.11)	-0.04 (-0.08, 0.0005)	0.053	0.085
IL2RA	22.1%	0.48 (0.48, 0.48)	-0.007 (-0.04, 0.02)	0.653	0.954

°The median of the 6 visits, #crude P-value, *adjusted for age and menstrual cycle.

Data on soluble inflammatory marker concentration were log-transformed before analysis. Analytes are reported as pg/ml. >LLD represents proportion of values above the lower limit of detection. Coefficient represents univariate analysis.

Supplementary Table S2: Impact of the genital microenvironment on the modulation of CD161 expression (Median Fluorescent Intensity - MFI) and $CD161^{+}CD4^{+}$ T cell recruitment in the FRT of HIV-negative FSWs from Nairobi, Kenya. Comparison of CD161 proportion and MFI between women infected or not (reference) with a genital bacterial infection (Bacterial Vaginosis –BV or sexually transmitted infections - STIs), at the follicular (reference) or the luteal phase of the menstrual cycle, or with low (25th percentile), middle (Interquartile range - IQR) or elevated level (75th percentile) genital concentration of inflammatory markers using linear regression and showing coefficient and confidence intervals (CI 95%). P-values were adjusted for age, or age and menstrual cycle.

	**% $CD161^{+}CD4^{+}$ T cells**			**MFI CD161**		
Predictors	Coefficient (CI 95%)	P-value[#]	P-value*	Coefficient (CI 95%)	P-value[#]	P-value*
Bacterial Vaginosis (BV) status						
Normal (reference)						
Intermediate	2.17 (-3.34, 7.69)	0.440	0.446	153.45 (-110.61, 417.50)	0.255	0.253
BV	0.35 (-5.49, 6.18)	0.908	0.668	142.21 (-134.39, 418.80)	0.314	0.221
Menstrual cycle phase						
Follicular						
Luteal	3.96 (-0.01, 7.93)	0.051	0.052[&]	164.42 (-24.89, 353.74)	0.089	0.089[&]
Sexually transmitted infection						
No STI (reference)						
Any STIs	7.62 (-4.19, 19.44)	0.206	0.241	358.35 (-203.91, 920.62)	0.212	0.246
Yeast infection						
Absence (reference)						
Presence	0.41 (-5.02, 5.84)	0.883	0.932	-76.30 (-333.35, 180.75)	0.561	0.544
Years of Sex Work						
< 1 year (reference)						
1-<3 year	2.05 (-11.49, 15.59)	0.767	0.964	-221.35 (-865.24, 422.54)	0.500	0.484
3-<7 year	-6.20 (-19.90, 7.51)	0.375	0.207	-177.41 (-826.30, 471.48)	0.592	0.606
7-15 year	3.33 (-9.24, 15.90)	0.603	0.911	64.06 (-530.03, 658.14)	0.833	0.883

> 15 year	-7.13 (-22.38, 8.11)	0.359	0.139	-233.14 (-952.86, 486.58)	0.525	0.533
Age						
age	0.17 (-0.28, 0.63)	0.455		1.34 (-20.37, 23.05)	0.904	
Genital Inflammatory markers						
IFN-g	1.13 (-2.76, 5.02)	0.568	0.473	286.13 (99.30-472.96)	**0.003**	**0.001**
IL-10	-1.68 (-6.44, 3.07)	0.488	0.535	40.21 (-147.35, 227.77)	0.674	0.488
IL12_p70	-0.56 (-3.60, 2.47)	0.716	0.976	124.92 (-15.93, 265.76)	0.082	0.037
sCD40L	-0.50 (-6.43, 5.43)	0.869	0.963	563.63 (287.10, 840.15)	**<0.0001**	**<0.0001**
IL-17A	1.53 (-2.56, 5.62)	0.463	0.337	139.51 (-27.63, 306.64)	0.102	0.116
IL-1a	0.37 (-2.96, 3.69)	0.829	0.959	233.16 (79.35, 386.96)	**0.003**	**0.006**
IL1b	-0.30 (-2.65, 2.21)	0.859	0.860	68.00 (-48.23, 184.22)	0.252	0.273
IL-2	0.39 (-3.61, 4.39)	0.847	0.709	168.74 (-21.59, 359.07)	0.082	0.062
IL-8	-0.90 (-3.56, 1.75)	0.504	0.526	8.52 (-121.81, 138.85)	0.898	0.896
IP-10	0.36 (-2.82, 3.55)	0.823	0.869	-30.24 (-179.28, 118.80)	0.691	0.609
MCP-1	1.55 (-4.54, 1.43)	0.308	0.644	-18.64 (-158.98, 121.70)	0.795	0.758
MIP-1a	-1.88 (-5.61, 1.86)	0.325	0.277	-7.31 (-182.98, 168.37)	0.935	0.826
MIP-1b	1.30 (-5.76, 3.16)	0.568	0.744	70.00 (-140.80, 280.80)	0.515	0.392
TNF-a	-3.31 (-7.11, 0.49)	0.088	0.107	98.99 (-80.27, 278.25)	0.279	0.252
IL1RA	0.70 (-2.86, 4.27)	0.699	0.531	71.82 (-101.93, 245.56)	0.418	0.365
MIG	1.05 (-1.49, 3.59)	0.418	0.517	76.18 (-43.38, 195.74)	0.212	0.346
MIP3a	-1.12 (-3.89, 1.64)	0.427	0.487	-32.42 (-162.85, 98.01)	0.626	0.725
IL2RA	-2.54 (-7.11, 2.03)	0.276	0.360	51.90 (-139.67, 243.47)	0.595	0.406

#crude P-value, *adjusted for age and menstrual cycle phase, or &age only.

Data on soluble inflammatory marker concentration were log-transformed before analysis. Analytes are reported as pg/ml. Coefficient represent univariate analysis. Sexually transmitted infections were grouped, “any STIs” includes two events *Neisseria gonorrhoea*, three events of *Chlamydia trachomatis*, one event of *Trichomonas vaginalis* and one event of syphilis).

Supplementary Table S3: Impact of Bacterial Vaginosis (BV) on the genital cytokine/chemokine concentrations in the FRT of HIV-negative FSWs from Nairobi, Kenya.

	**BV Infection (normal, reference)**					
	Coefficient (CI 95%)		P-value#		P-value*	
Genital Inflammatory markers (log_{10} pg/ml)	Intermediate	BV	Intermediate	BV	Intermediate	BV
IFN-g	0.11 (-0.02, 0.25)	0.30 (0.15, 0.45)	0.107	<0.001	0.119	**<0.001**
IL-10	0.13 (0.01, 0.27)	0.21 (0.07, 0.34)	0.037	0.004	**0.035**	**0.004**
IL12_p70	0.23 (0.05, 0.42)	0.49 (0.29, 0.69)	0.014	<0.001	**0.017**	**<0.001**
sCD40L	0.07 (-0.01, 0.16)	0.18 (0.09, 0.27)	0.087	<0.001	0.087	**<0.001**
IL-17A	0.13 (-0.03, 0.29)	0.28 (0.11, 0.46)	0.107	0.001	0.113	**0.003**
IL-1a	0.32 (0.16, 0.48)	0.49 (0.31, 0.67)	<0.001	<0.001	**<0.001**	**<0.001**
IL1b	0.62 (0.40, 0.85)	0.87 (0.63, 1.11)	<0.001	<0.001	**<0.001**	**<0.001**
IL-2	0.07 (-0.07, 0.20)	0.24 (0.10, 0.39)	0.334	0.001	0.364	**0.002**
IL-8	-0.04 (-0.25, 0.16)	0.07 (-0.15, 0.29)	0.663	0.552	0.626	0.614
IP-10	-0.44 (-0.62, -0.27)	-0.49 (-0.68, -0.30)	<0.001	<0.001	**<0.001**	**<0.001**
MCP-1	-0.31 (-0.52, -0.11)	-0.25 (-0.47, -0.05)	0.002	0.017	**0.001**	**0.003**
MIP-1a	0.04 (-0.11, 0.19)	0.12 (-0.05, 0.28)	0.575	0.163	0.596	0.142
MIP-1b	0.02 (-0.10, 0.15)	0.09 (-0.05, 0.22)	0.740	0.202	0.792	0.354
TNF-a	0.22 (0.08, 0.35)	0.29 (0.14, 0.43)	0.002	<0.001	**0.002**	**<0.001**
IL1RA	0.22 (0.06, 0.38)	0.11 (-0.06, 0.29)	0.006	0.187	**0.007**	0.224
MIG	-0.54 (-0.75, -0.33)	-0.72 (-0.96, -0.49)	<0.001	<0.001	**<0.001**	**<0.001**
MIP3a	-0.19 (-0.38, 0.01)	-0.21 (-0.42, -0.001)	0.066	0.049	0.065	0.054
IL2RA	0.10 (-0.05, 0.26)	0.18 (0.01, 0.34)	0.199	0.035	0.198	0.052

#crude P-value, *adjusted for age and menstrual cycle phase.

Data on soluble inflammatory marker concentration were log-transformed before analysis. Analytes are reported as pg/ml. Coefficient represent univariate analysis.
